# Differences in Muscle Metabolism Between Triathletes and Normally Active Volunteers Investigated Using Multinuclear Magnetic Resonance Spectroscopy at 7T

**DOI:** 10.3389/fphys.2018.00300

**Published:** 2018-04-03

**Authors:** Radka Klepochová, Ladislav Valkovič, Thomas Hochwartner, Christoph Triska, Norbert Bachl, Harald Tschan, Siegfried Trattnig, Michael Krebs, Martin Krššák

**Affiliations:** ^1^Department of Biomedical Imaging and Image-Guided Therapy, High-Field MR Center, Medical University of Vienna, Vienna, Austria; ^2^Christian Doppler Laboratory for Clinical Molecular MR Imaging, MOLIMA, Vienna, Austria; ^3^Oxford Centre for Clinical Magnetic Resonance Research, BHF Centre of Research Excellence, University of Oxford, Oxford, United Kingdom; ^4^Department of Imaging Methods, Institute of Measurements Science, Slovak Academy of Sciences, Bratislava, Slovakia; ^5^Centre of Sport Science and University Sport, University of Vienna, Vienna, Austria; ^6^Division of Endocrinology and Metabolism, Department of Internal Medicine III, Medical University of Vienna, Vienna, Austria

**Keywords:** proton and phosphorus magnetic resonance spectroscopy, skeletal muscle, oxygen uptake, muscle training status, energy metabolism

## Abstract

**Purpose:** The influence of endurance training on skeletal muscle metabolism can currently be studied only by invasive sampling or through a few related parameters that are investigated by either proton (^1^H) or phosphorus (^31^P) magnetic resonance spectroscopy (MRS). The aim of this study was to compare the metabolic differences between endurance-trained triathletes and healthy volunteers using multi-parametric data acquired by both, ^31^P- and ^1^H-MRS, at ultra-high field (7T) in a single experimental protocol. This study also aimed to determine the interrelations between these MRS-derived metabolic parameters.

**Methods:** Thirteen male triathletes and ten active male volunteers participated in the study. Proton MRS data from the vastus lateralis yielded concentrations of acetylcarnitine, carnosine, and intramyocellular lipids (IMCL). For the measurement of phosphodiesters (PDEs), inorganic phosphate (Pi), phosphocreatine (PCr), and maximal oxidative capacity (Q_max_) phosphorus MRS data were acquired at rest, during 6 min of submaximal exercise and following immediate recovery.

**Results:** The triathletes exhibited significantly higher IMCL levels, higher initial rate of PCr resynthesis (V_PCr_) during the recovery period, a shorter PCr recovery time constant (τ_PCr_), and higher Q_max_. Multivariate stepwise regression analysis identified PDE as the strongest independent predictor of whole-body maximal oxygen uptake (VO_2max_).

**Conclusion:** In conclusion, we cannot suggest a single MRS-based parameter as an exclusive biomarker of muscular fitness and training status. There is, rather, a combination of different parameters, assessable during a single multi-nuclear MRS session that could be useful for further cross-sectional and/or focused interventional studies on skeletal muscle fitness and training effects.

## Introduction

The main tissue responsible for whole-body energy metabolism, glycogen storage, and glucose uptake, is skeletal muscle (Defronzo et al., 1981; DeFronzo and Tripathy, 2009). It is well-known that endurance training alters functional demands in skeletal muscle (Fujimoto et al., 2003). It has been shown that athletes with a background of endurance training have better oxidative metabolism (Pette, 1998), improved glucose uptake (Fujimoto et al., 2003), as well as enhanced capillarity and increased mitochondrial density (Pette, 1998). Moreover, the potential of exercise to adapt oxidative potential of skeletal muscles has been demonstrated by increased succinate dehydrogenase activity mainly in actively involved muscle fibers, i.e., slow-twitch muscle fibers which are primarily effected by endurance training (Saltin et al., 1977). Endurance training and long lasting low-load resistance exercise also generate metabolic stress, that can accumulate into adaptation in mitochondrial content and function (Groennebaek and Vissing, 2017). The vast majority of these observations are the result of biopsies or *ex vivo* measurements, unsuitable for repetitive assessment of skeletal muscle status during the training process.

Magnetic resonance spectroscopy (MRS) is a non-invasive alternative technique bound to define the performance-related characteristics of skeletal muscle and to explore the differences between skeletal muscle of endurance-trained men and untrained participants. Especially, proton (^1^H) and phosphorus (^31^P) MRS has been used to provide information about the metabolism of a tissue.

In particular, it has been reported that the carnosine content, which is measurable by localized ^1^H-MRS, could potentially be a good indicator for estimating muscle fiber composition, as it is present in different concentrations in slow-twitch than in fast-twitch fibers (Baguet et al., 2011). Further, a well-studied phenomenon in the context of skeletal muscle metabolism and athletic performance, also detectable by ^1^H-MRS, are intramyocellular lipids (IMCL). Usually, elevated IMCL levels can be found in the sedentary population, and, in the majority of cases, suggest impaired glucose uptake, which is closely related to insulin resistance (Krššák et al., 1999) and contributes to the development of obesity and type 2 diabetes mellitus (T2DM) (Kautzky-Willer et al., 2003). However, high IMCL levels alone cannot be taken as a sign of a lipid metabolism defect, as endurance-trained athletes have shown high IMCL concentrations without metabolic impairment (Thamer et al., 2003; Dubé et al., 2008). Another metabolite assessable by muscle ^1^H-MRS, acetylcarnitine, is important for maintaining pyruvate dehydrogenation activity, known to control the rate of aerobic carbohydrate oxidation (Constantin-Teodosiu, 2013). Moreover, skeletal muscle acetylcarnitine concentration correlates negatively with insulin resistance, with highest fasting concentrations of acetylcarnitine observed in endurance-trained athletes and the lowest in T2DM patients (Lindeboom et al., 2014).

Furthermore, ^31^P-MRS allows direct measurement of energy-rich metabolites, i.e., phosphocreatine (PCr), adenosine-tri-phosphate (ATP), and can be used dynamically to investigate oxidative energy production by mitochondria during exercise and subsequent recovery (Kemp and Radda, 1994; Kemp et al., 2015; Valkovič et al., 2017). Dynamic ^31^P-MRS has been used to probe human muscle metabolism since the 1980's (Chance et al., 1980; Meyer and Brown, 1982) with first *in vivo* studies focused on assessment of steady-state capability of oxidative phosphorylation of forearm muscle using the PCr/Pi ratio and dynamic changes in intracellular pH (Chance et al., 1981; Taylor et al., 1983; Arnold et al., 1984). During the years, PCr recovery rate after exercise has taken over as a measure of mitochondrial oxidative production rate (Boska, 1991; Walter et al., 1999; Kemp et al., 2015). Endurance trained athletes have been shown to recover PCr after exercise faster than untrained (McCully et al., 1989; Layec et al., 2009) and even sprint-trained athletes (Crowther et al., 2002; Johansen and Quistorff, 2003) reflecting their superior oxidative metabolism function.

Alternatively, the content of elevated skeletal muscle phosphodiesters (PDEs), measured by ^31^P-MRS, in age-matched participants, was suggested as a potential marker of muscle oxidative capacity (Valkovič et al., 2016). Changes in muscle mitochondrial density in response to training can potentially be assessed through alkaline inorganic phosphate (Pi) pool (Pi_2_) concentrations or Pi_2_/Pi ratios (Kan et al., 2010; Valkovič et al., 2016). In addition, ^31^P-MRS saturation transfer techniques allow estimation of PCr-to-ATP and Pi-to-ATP fluxes (F_CK_ and F_ATP_, respectively) even in muscles at rest (Valkovič et al., 2017). These resting measurements correlate with the findings of dynamic experiments (Schmid et al., 2012; Valkovič et al., 2016) and have been related to insulin resistance (Petersen et al., 2004). Nevertheless, the absolute values of F_ATP_ measured at rest significantly overestimates pure oxidative ATP synthesis, mainly due to strong glycolytic component of F_ATP_ and close to equilibrium turnover at rest (Balaban and Koretsky, 2011; From and Ugurbil, 2011). On the other hand, if measured during steady-state aerobic exercise, the glycolytic component of F_ATP_ will be minimal and the total measured flux will represent mitochondrial ATP turnover more closely (Brindle et al., 1989).

Taken together, ^31^P and ^1^H-MRS both provide ideal non-invasive tools for the *in vivo* assessment of skeletal muscle metabolism, which plays an essential role in whole-body energy metabolism and is the key factor in athletic performance. However, the applicability of different MRS methods on clinical lower-field MR systems has been limited by insufficient spectral resolution and rather low spectral and experimental signal-to-noise-ratio, which resulted in relatively long acquisition times, making a comprehensive measurement unsuitable for monitoring of muscular training status, i.e., the metabolic fitness of a skeletal muscle, or for routine examinations during the training process.

MRS examinations at ultra-high field (7T) provide, generally, better spectral resolution, higher repeatability, and improvements that have led to the opportunity to measure subtle signals of parameters for energy metabolism [i.e., carnosine, acetylcarnitine, the alkaline Pi pool (Pi_2_), PDE, i.e., glycerol-phosphocholine (GPC), and glycerol-phosphoethanolamine (GPE)], and to assess metabolic reaction rates under steady-state exercise conditions, which have never been measured in athletes in one integrative study.

Our aim was to use multi-nuclear MRS at 7T to compare the value of suggested basal and dynamic markers of oxidative metabolism in different training status of vastus lateralis (VL) in two groups of volunteers: healthy active individuals and endurance-trained triathletes. In our measurement and analysis we included (i) the examination of IMCL content in the context of the paradox of an athlete's high IMCL levels, (ii) the assessment of acetylcarnitine concentration because of its important role in lipid metabolism, and (iii) and the measurement carnosine concentration as a possible consequence of muscle fiber-type distribution. Moreover, endurance-trained athletes should have a higher volume of mitochondrial density, and, therefore, faster oxidative metabolism and faster PCr resynthesis following submaximal exercise (Johansen and Quistorff, 2003; Bogdanis, 2012). New, recently suggested markers of oxidative muscle metabolism, e.g., alkaline Pi, PDE, and Pi-to-ATP reaction rate measured in metabolic steady state during aerobic exercise, were also compared between the groups. As a final point we aimed to perform a bivariate and multivariate analysis of the interrelations between measured metabolic parameters.

## Methods

### Study population

Thirteen male triathletes (age = 28.2 ± 5.7 years, BMI = 22.3±1.4 kg.m^−2^) and 10 healthy active male volunteers (age = 26.2 ± 4.7 years, BMI = 22.8 ± 2.6 kg.m^−2^) participated in the study.

All participating athletes were in a pre-season preparation phase without any special training dedicated methods, and without special diet or nutrition supplementation. All of them have been competing in triathlon events across various distances for at least 3 years at a national level, and the performance criterion was the time they needed to cycle 20 km (maximum allowed: 39 min) and 40 km (maximum allowed: 70 min) during their past competitions.

For the group of active volunteers, the inclusion criterion was normal and regular physical activity, e.g., cycling, running, gym, and team sports up to three times per week. No special nutrition and supplementation or any physical activity, such as weightlifting or special strength training, was required as an inclusion factor.

All participants, triathletes and healthy active volunteers, refrained from any physical activity 24 h before MR examinations and had no other changes in daily routine. Written, informed consent was provided before commencing and the study protocol adhered to the local ethics committee requirements.

One week before MR examinations, all participants underwent a standardized protocol for the determination of maximal oxygen uptake (VO_2max_) on a bicycle ergometer (Lode Excalibur, Groningen, The Netherlands) with continuous increments, until exhaustion, Measurement of VO_2max_ was performed via “breath-by-breath” spiroergometry (Master CPX, VIASYS Healthcare). The test protocol included a 3-min adaptation phase for the measurement of the baseline and subsequent continuous increases of 20 W/min with a constant RPM of 90 rpm up to the individual maximal exhaustion. This ensured that the total duration of the spiroergometry for all subjects (trained and untrained) was between 7 min and a maximum of 26 min.

### Magnetic resonance (MR) measurements

The entire MR examination protocol was performed within a single visit, starting at the same time in the afternoon, approximately 1 h after a mixed meal lunch. All MR measurements were performed on a 7T whole-body MR system (Siemens Healthcare, Erlangen, Germany). A 28-channel knee coil (QED, Mayfield Village, OH, USA) was used to acquire ^1^H-MR spectra and a dual-tuned (^31^P/^1^H) circular surface coil (10 cm diameter, Rapid Biomedical, Rimpar, Germany) was used to acquire ^31^P-MR spectra from the quadriceps of the left leg.

**Figure 1:**
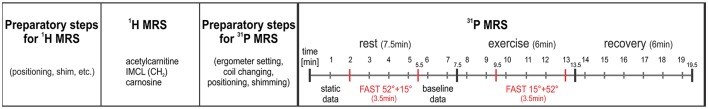
Schematic representation of the measurement protocol. First, volunteers were in the supine position with the left thigh muscle placed inside the 28-channel knee coil for the acquisition of the ^1^H-MRS data. Afterward, the volunteers turned into the prone position, for the ^31^P-MRS measurement, and their thigh muscle was strapped onto an MR-compatible ergometer dedicated for knee extension exercise. The ^31^P-MRS protocol consisted of 7.5 min of measurement at rest (2 min of partially relaxed, long TR static data acquisition, 3.5 min of four-angle saturation transfer (FAST) and 2 min of baseline data for the dynamic experiment) followed by 6 min of knee extension exercise (including the 3.5 min long FAST measurement starting 2 min after the onset of exercise, i.e., once the steady-state is reached) and ending with 6 min of recovery.

The measurements were divided into two parts. First, volunteers were in the supine position with the left thigh muscle placed inside the 28-channel knee coil for the acquisition of the ^1^H-MRS data. Afterward, the volunteers turned into the prone position, for the ^31^P-MRS measurement, and their thigh muscle was strapped onto an MR-compatible ergometer (Quadspect, Ergospect, Innsbruck, Austria) dedicated for knee extension exercise. The exercise resistance was set to 30% of the maximal voluntary contraction (MVC) force, determined by repeated measurement of the maximum force generated against the pedal fixed in the exercise position. The ^1^H/^31^P surface coil was positioned under the thigh muscles of the left leg. The localized ^31^P-MRS data acquisition which is described below in detail was performed before and during exercise and recovery protocol. This consisted of 7.5 min of baseline period dedicated for measurement at rest followed by 6 min of knee extension exercise and ending with 6 min of recovery (Valkovič et al., 2014; Tušek Jelenc et al., 2016). The volunteers were instructed by an audio signal to time the contraction-relaxation periods (exercise frequency 0.5 Hz), so that the spectra were always acquired during the relaxed state of the muscle under investigation. A schematic illustration of the experimental design is shown in Figure 1.

#### ^1^H-MRS

T_1_ weighted, multi-slice localizer images were acquired and used for volume-of-interest (VOI) positioning. Spatial selection was achieved using a STEAM localization sequence and the VOI of 21 ml (40 × 35 × 15 mm^3^) was carefully placed in the VL muscle. Voxel positioning was guided by images so as to properly adjust voxel geometry and orientation to fit the actual shape of subject's muscle, avoiding the boundaries of muscle and subcutaneous fat. Localized shimming was performed manually, on the adjustment volume that matched the VOI, after automatic field-map acquisition based on gradient recalled, double echo field-map acquisition (GRE-SHIM, Siemens Healthcare, Erlangen, Germany). The final linewidth of the water signal was in the range of 28–38 Hz in the magnitude mode.

Signals of acetylcarnitine and IMCL were measured with the following parameters: repetition time (TR)/echo time (TE) = 2,000/350 ms; spectral bandwidth = 3 kHz; number of averages (NA) = 128; delta frequency = −2.5 ppm relative to water resonance; number of preparation scans = 4 (Klepochová et al., 2017).

The MR signal of carnosine was measured with water signal suppression and the following parameters: TR/TE = 9,000/20 ms; spectral bandwidth = 3 kHz; NA = 64; delta frequency = 2.8 ppm relative to water resonance; number of preparation scans = 0, as described by Just Kukurová et al. (2016).

For acquisition of a concentration reference, the water signal was measured with a TR = 2,000 ms and a TE = 20 ms; NA = 1; and delta frequency = 0 ppm.

#### ^31^P-MRS

Depth-resolved *in vivo* spectroscopy (DRESS) localization (Bottomley et al., 1984) was used for ^31^P-MRS signal detection at rest, as well as during exercise and recovery. In particular, a 15 mm-thick MRS selection slab representing the VOI was placed over the VL, based on localizer images, and manual shimming was performed. A 90° FA was adjusted by finding the RF transmit voltage that provided the maximum of the localized PCr signal. No decoupling or NOE was used.

During the resting baseline, the assessment of metabolite concentrations static long TR ^31^P MR spectra (FA = 90°, TR = 15 s, NA = 8, acquisition time = 2 min) was performed. This measurement was followed by DRESS-localized four-angle saturation transfer (FAST) measurements performed in two experiments during next 3.5 min of resting baseline. The first part applied nominal FA of 52° (β) and NA = 8, and the second part applied nominal FA of 15° (α) and NA = 24, as described previously (Tušek Jelenc et al., 2016). The FAST measurement at rest was followed by the dynamic protocol with temporal resolution of 2 s, i.e., single shot acquisition with TR = 2 s. The dynamic protocol consisted of 2 min for baseline data, 6 min of knee extension exercise and 6 min of recovery (Valkovič et al., 2014). During exercise-induced metabolic steady-state, the FAST experiment was repeated to assess the reactions of creatine kinase (CK) and the ATP synthesis/hydrolysis cycle, as described previously (Tušek Jelenc et al., 2016). During the last 30 s of exercise and during the following recovery localized acquisition of dynamic ^31^P MRS data was performed again. Overview about the timing of ^31^P MRS data acquisition is given also in the Figure 1.

At the end our comprehensive dynamic ^31^P MRS protocol yielded basal concentrations of phosphorus containing metabolites at rest, forward rate of oxidative ATP synthesis (F_ATP_) and creatine kinase (F_CK_) at rest and during exercise, depletion of PCr during the exercise, time constant and rate of PCr resynthesis during recovery (τ_PCr_, V_PCr_), maximal rate of oxidative phosphorylation, i.e., mitochondrial capacity (Q_max_), and time course of intracellular pH changes in skeletal muscle.

### Data processing, calculations, and statistical analysis

All measured spectra were analyzed using the jMRUI (Java-Based Magnetic Resonance User Interface; version 5) with the AMARES (Advanced Method for Accurate, Robust, and Efficient Spectral Fitting) time-domain fitting algorithm (Vanhamme et al., 1997).

#### ^1^H-MRS

Spectral lines of acetylcarnitine, carnosine, IMCL, and water were fitted as a single Lorentzian. Lipid signals surrounding the acetylcarnitine peak were fitted with a constrained frequency to avoid their influence on fitted acetylcarnitine (Klepochová et al., 2017).

The C_2_-H peak of carnosine, resonating at 8 ppm, was fitted after removing residual peaks of water and lipids by a Hankel Lanczos Squares Singular Values Decomposition (HSVLD) algorithm, as it was shown to be the more robust of two available carnosine signals (Just Kukurová et al., 2016).

Using the water signal as an internal reference, the concentration of acetylcarnitine and carnosine were calculated according to the formula for millimolar concentrations in 1 kg of wet weight of tissue (mmol/kg ww):

$$C_{\text{m}}={C_{\text{H}2\text{O}}}^{*}{\left(S_{\text{m}}/S_{\text{H}2\text{O}}\right)}^{*}{\left(n_{\text{H}2\text{O}}/n_{\text{m}}\right)}^{*}{\left(CF_{\text{H}2\text{O}}/CF_{\text{m}}\right)}^{*}w_{\text{H}2\text{O}}$$

where S is the signal intensities of H_2_O-water, m is the metabolite (e.g., acetylcarnitine or carnosine), n_H2O_ is the number of corresponding equivalent protons in water (*n* = 2) and n_m_ is for the acetylcarnitine (*n* = 3) or carnosine (*n* = 1) molecule, CF is the correction factors for T_1_ and T_2_ relaxations, C_H2O_ = 55.56 mol/L is the concentration of the water, and w_H2O_ is the approximate water content of skeletal muscle tissue, i.e., 0.77 L/kg wet weight of tissue.

IMCL CH_2_ (methylene) at 1.3 ppm was fitted, corrected for T_1_ and T_2_ relaxation effects, and expressed as a percentage of water content.

Literature relaxation times were used for T_1_ and T_2_ corrections (Bottomley et al., 1984; Ren et al., 2011; Just Kukurová et al., 2016).

#### ^31^P-MRS

The resonance lines of PCr, Pi, Pi_2_, glycerol-phosphocholine (GPC), and glycerol-phosphoethanolamine (GPE) were fitted as single Lorentzians, whereas γ- and α-ATP were fitted as doublets and β-ATP as a triplet. The concentration of phosphodiesters (PDEs) was calculated as the sum of GPC and GPE. The frequency of the Pi_2_ peak was constrained with respect to the expected frequency difference between Pi_2_ and Pi to ~0.4 ppm (Kan et al., 2010). For the FAST experiment, the linewidths of PCr and Pi were determined from the highest signal-to-noise ratio spectra and then constrained to ±5 Hz (Tušek Jelenc et al., 2016; Valkovič et al., 2016). The apparent relaxation times (${\text{T}}_{1}^{\text{app}}$) and pseudo first-order exchange rate constants of the CK reaction (k_CK_) and ATP synthesis reaction (k_ATP_) were calculated according to the equations for the FAST experiment (Bottomley et al., 2002). For the quantification of metabolite concentrations, a cellular γ-ATP concentration of 8.2 mM was assumed (Taylor et al., 1986). Forward metabolic fluxes (F_ATP_, F_CK_) were the product of k_ATP_ and k_CK_ times the concentration of Pi and PCr, respectively (Valkovič et al., 2017).

The shift in resonance position between PCr and Pi signals was used to calculate intramyocellular pH (Moon and Richards, 1973) according to the modified Henderson-Hasselbalch equation. ADP concentration was calculated according to the modified method described by Kemp et al. (1993b), assuming that 15% of total creatine was not phosphorylated in the resting state.

To calculate the time constant of PCr resynthesis (τ_PCr_), the PCr signal changes during the recovery period of the dynamic experiment were fitted to a mono-exponential function. This was used to calculate the initial PCr recovery rate (V_PCr_), which roughly represents ATP turnover at the end of exercise and the maximal rate of oxidative phosphorylation, i.e., mitochondrial capacity (Q_max_), was calculated according to the ADP-based model of Michaelis and Menten (Kemp et al., 1993b; Valkovič et al., 2016).

### Statistical analysis

Data are presented as means ± standard deviations and were compared between the two groups by an unpaired Student's *t-*test, controlling for multiple comparisons using Benjamini–Hochberg procedure (Benjamini and Hochberg, 1995). The relationships between metabolic parameters determined using ^1^H and ^31^P-MRS were analyzed by linear correlations using Pearson's correlation coefficient (two-tailed probability values) to estimate the strength of the relationship. The correlation coefficient of an absolute value of 0.415, which corresponded to a 95% confidence agreement, was taken as significant. A stepwise regression analysis for the dependent variable, VO_2max_, was performed using the independent variables (i.e., τ_PCr_, V_PCr_, Q_max_, PDE, GPC, IMCL, acetylcarnitine). The results were considered statistically significant at *p* < 0.05 (*t*-test) with Benjamini–Hochberg adjusted *p* < 0.25.

## Results

### Skeletal muscle metabolism of triathletes

As expected triathletes presented with a significantly higher maximal aerobic capacity (VO_2max_; *p* < 0.001) when compared to healthy active volunteers.

**Figure 2:**
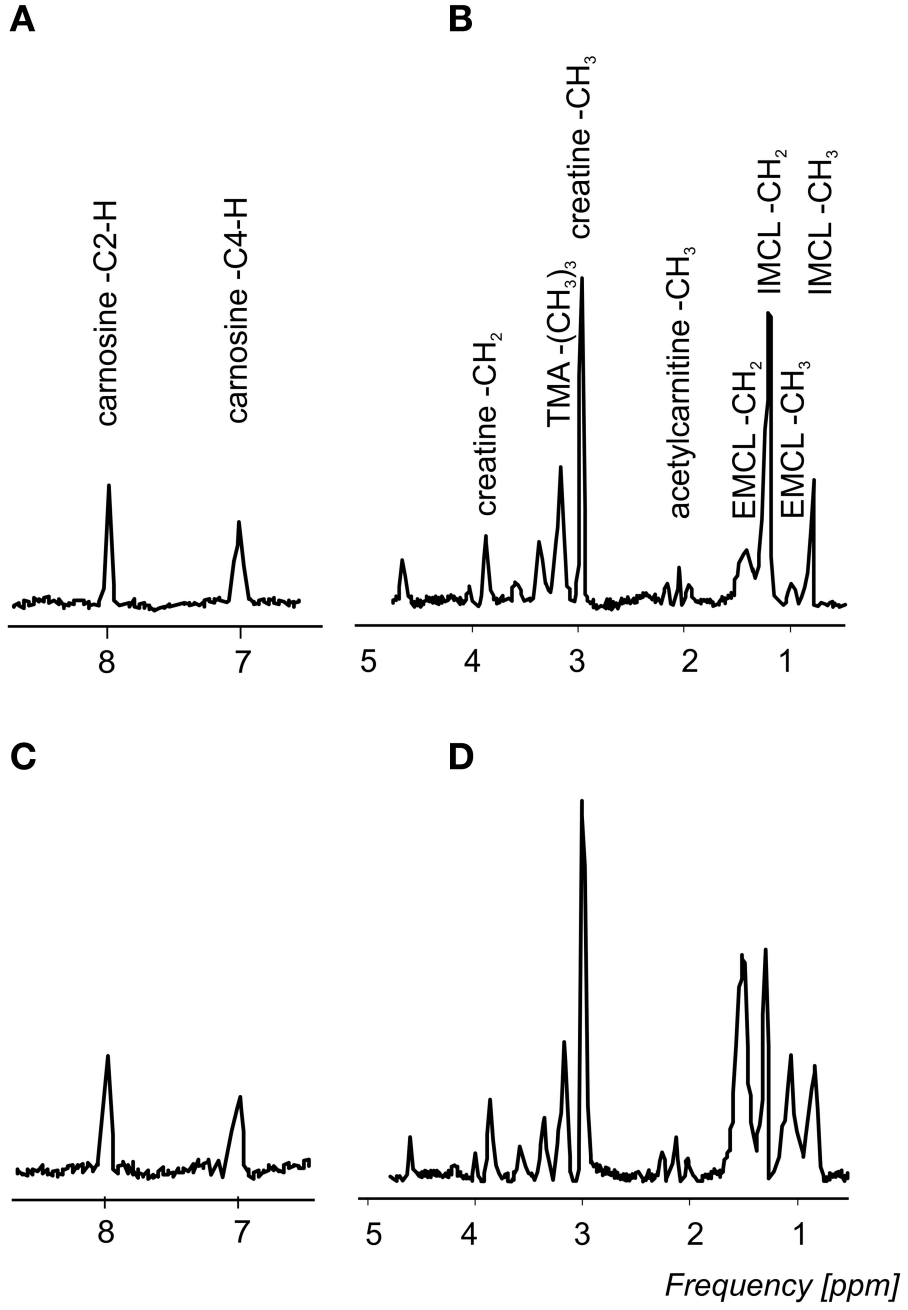
A representative ^1^H-MRS spectrum from a triathlete **(A,B)** and a healthy active volunteer **(C,D)** acquired from the vastus lateralis muscle. **(A,C)** present carnosine spectral lines at 7 and 8 ppm and **(B,D)** show the trimethyl ammonium (TMA) groups of carnitine, acetylcarnitine, and choline at 3.20 ppm, creatines at 3.03, and 3.9 ppm, acetylcarnitine at 2.13 ppm, and extramyocellular (EMCL) and intramyocellular (IMCL) lipid resonance lines at 1.5 and 1.3 ppm (CH_2_ group). For better visualization, a 1.5-Hz Lorentzian apodization filter was applied to reduce noise in the spectra. All spectra were normalized with respect to the water signal.

**Figure 3:**
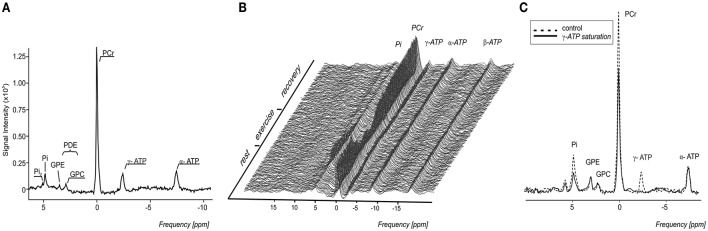
**(A)** A representative highly spectrally resolved ^31^P-MRS spectra. For better visualization, a 1,5-Hz Lorentzian apodization filter was applied to reduce noise in the spectra. **(B)** Time course of a ^31^P MR spectra during a knee extension exercise with depicted depletion of the PCr signal and its subsequent re-synthesis during the recovery period. **(C)** Saturation transfer spectra showing the effect of γ-ATP saturation, at ~-2.48 ppm (solid line) on the Pi signal compared to the control experiment with saturation at ~12.52 ppm (dashed line).

**Figure 4:**
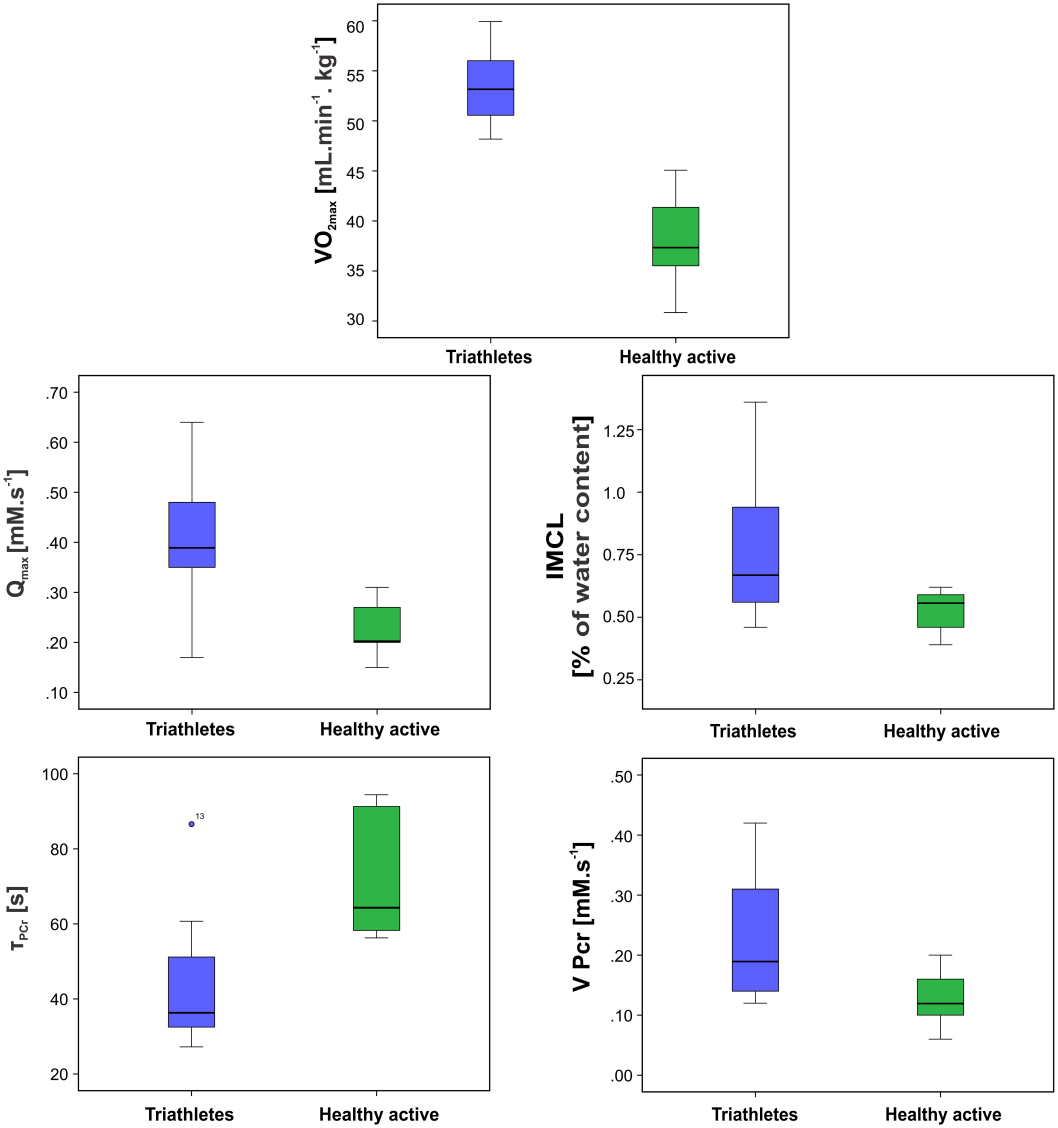
Box plots depicting the significantly different parameters between the two groups. The solid lines represent the median, boxes represent lower and upper quartiles, and whiskers the minimum and maximum. Outliers are denoted by circles. The outliers were also taken into account for all statistical tests.

Typical ^1^H-MR spectra acquired in a triathlete and normally active volunteer, are depicted in Figure 2 and representative ^31^P-MR spectra acquired at rest, during the exercise-recovery experiment, and during the FAST experiment are depicted in Figure 3. Triathletes differed in several MRS-derived metabolic read-outs. In particular, the triathlete group exhibited a significantly higher IMCL (*p* = 0.009), higher initial rate of PCr resynthesis (V_PCr_) during the recovery period (*p* = 0.006), shorter PCr recovery time constant (τ_PCr_) (*p* = 0.0007), and higher maximal oxidative flux (Q_max_) (*p* = 0.0003). Detailed results of both participating study groups are displayed in Figure 4 and listed in Table 1.

**Table 1:** Results from ^1^H and ^31^P-MRS and spiroergometry.

	**Triathletes (*n* = 13)**	**Healthy active (*n* = 10)**	***t-*test *p-*values (Benjamini–Hochberg *p*-values)**
Age (years)	28 ± 6	26 ± 5	
BMI (kg.m^−2^)	22.3 ± 1.4	22.8 ± 2.6	
VO_2max_ (mL.min^−1^. kg^−1^)	53.7 ± 4.1	37.8 ± 4.1	**<0.001 (<0.001)**
^1^**H-MRS**			
[Acetylcarnitine] (mmol/kg ww)	1.75 ± 0.94	1.61 ± 1.09	0.795 (0.869)
[Carnosine] (mmol/kg ww)	3.82 ± 1.10	3.12 ± 0.67	0.838 (0.889)
[IMCL CH_2_] (% of water resonance peak intensity)	0.78 ± 0.28	0.52 ± 0.11	**0.009 (0.066)**
^31^**P-MRS STATIC**			
[PCr] _at rest_ (mM)	28.6 ± 3.6	26.7 ± 3.7	0.239 (0.464)
[Pi] _at rest_ (mM)	3.69 ± 0.61	3.37 ± 0.6	0.162 (0.379)
[GPC] (mM)	2.27 ± 0.58	3.05 ± 1.35	0.110 (0.350)
[GPE] (mM)	0.64 ± 0.35	0.81 ± 0.35	0.255 (0.469)
[PDE] (mM)	2.91 ± 0.77	3.87 ± 1.24	0.049 (0.252)
[Pi_2_] (mM)	0.46 ± 0.24	0.42 ± 0.13	0.548 (0.685)
Pi_2_/Pi ratio	0.13 ± 0.08	0.12 ± 0.04	0.764 (0.862)
pH _rest_	7.10 ± 0.02	7.10 ± 0.03	0.470 (0.685)
^31^**P-MRS FAST AT REST**			
k_ATP_ (s^−1^)	0.11 ± 0.05	0.10 ± 0.04	0.549 (0.708)
F_ATP_ (mM.s^−1^)	0.48 ± 0.20	0.39 ± 0.16	0.524 (0.685)
k_CK_ (s^−1^)	0.27 ± 0.07	0.23 ± 0.04	0.058 (0.252)
F_CK_ (mM.s^−1^)	7.90 ± 1.90	7.16 ± 1.54	0.316 (0.554)
^31^**P-MRS FAST DURING EXERCISE**			
k_ATP_ (s^−1^)	0.08 ± 0.04	0.06 ± 0.03	0.541 (0.685)
F_ATP_ (mM.s^−1^)	0.85 ± 0.39	0.72 ± 0.44	0.525 (0.685)
k_CK_ (s^−1^)	0.13 ± 0.08	0.15 ± 0.03	0.525 (0.685)
F_CK_ (mM.s^−1^)	2.37 ± 0.89	3.22 ± 1.03	0.052 (0.252)
^31^**P-MRS DYNAMIC**			
τ_PCr_ (s)	46.45 ± 15.73	72.26 ± 16.44	**<0.001 (0.009)**
V_PCr_ (mM.s^−1^)	0.23 ± 0.11	0.13 ± 0.05	**0.006 (0.057)**
Q_max_ (mM.s^−1^)	0.41 ± 0.13	0.23 ± 0.05	**<0.001 (0.006)**
PCr drop (%)	33.91 ± 16.12	34.02 ± 11.53	0.986 (0.986)
[PCr] _end of exercise_ (mM)	19.71 ± 5.89	18.22 ± 5.05	0.521 (0.685)
[Pi] _end of exercise_ (mM)	8.37 ± 2.86	6.79 ± 3.17	0.231 (0.464)
pH _end of exercise_	7.05 ± 0.03	7.03 ± 0.02	0.099 (0.350)
[ADP] _end of exercise_ (μM)	46.58 ± 28.52	42.39 ± 18.22	0.673 (0.785)

*Data are given as mean ± standard deviation. The p-values are denoted in the last column. Statistical significance (marked in bold) was reached for t-test p < 0.05 and Benjamini–Hochberg adjusted p < 0.25*.

### Correlations between the measured parameters

Bivariate correlation analysis of pooled data revealed, that VO_2max_ was positively correlated with Q_max_ (*r* = 0.66, *p* = 0.0006), V_PCr_ (*r* = 0.48, *p* = 0.02), and PCr concentration measured at the end of recovery (*r* = 0.48, *p* = 0.02), and negatively with τ_PCr_ (*r* = −0.64, *p* = 0.001), PDE (*r* = −0.57, *p* = 0.004), and GPC (*r* = −0.48, *p* = 0.02). GPC, and PDE were positively correlated with BMI (both, *r* = 0.54, *p* = 0.007), and, moreover, GPC was positively correlated with age (*r* = 0.44, *p* = 0.03).

**Figure 5:**
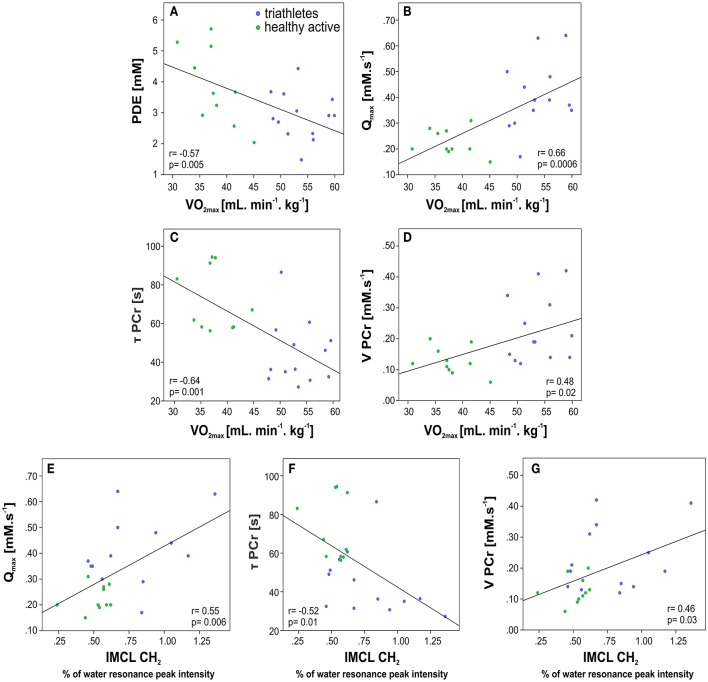
Plots of correlations between parameters measured by spiroergometry and ^31^P-MRS in triathletes and healthy active volunteers: **(A)** maximum oxygen uptake (VO_2max_) with the phosphodiesters (PDE); **(B)** VO_2max_ with mitochondrial capacity (Q_max_); **(C)** VO_2max_ with PCr recovery rate time constant (τ_PCr_); **(D)** VO_2max_ with initial rate of PCr resynthesis (V_PCr_) during the recovery period; and between the parameters measured by ^1^H- and ^31^P-MRS: **(E)** intramyocellular lipid content (IMCL) with Q_max_; **(F)** IMCL with τ_PCr_ and **(G)** IMCL with V PCr.

Several correlations were also found between the metabolic parameters extracted from the ^31^P-MRS measurements and the parameters from ^1^H-MRS, specifically acetylcarnitine was negatively correlated with GPC and PDE (*r* = −0.43, *p* = 0.04). IMCL was negatively correlated with τ_PCr_ (*r* = −0.52, *p* = 0.01), and positively with Qmax (*r* = 0.55, *p* = 0.006) and V_PCr_ (*r* = 0.46, *p* = 0.02). Representative bivariate correlations are depicted in Figure 5.

Multivariate stepwise regression analysis identified [PDE] (*p* = 0.004) as the strongest independent predictor of VO_2max_.

## Discussion

The purpose of this study was to investigate the value of different MRS-derived read-outs of oxidative skeletal muscle metabolism for the prediction of whole body maximal oxygen uptake in endurance trained athletes and healthy active volunteers by non-invasive multi-nuclear MRS. The measurements were performed on vastus lateralis muscle, as this muscle is most representative for whole body oxygen uptake during cycling and it is mostly frequently studied region of skeletal musculature in men in the past. We present combined results and correlation analysis from localized, static ^1^H-MRS and static as well as dynamic ^31^P-MRS, measured in one experimental protocol for the first time.

The most important intergroup findings in our study were: In the triathlete group, in line with a 42% higher VO_2max_, we found a 50% higher IMCL content, 36% faster PCr resynthesis (τ_PCr_), 76% higher V_PCr_, and a 78% higher mitochondrial capacity (Q_max_) in vastus lateralis muscle. Besides, even though there was no significant difference in PDE found in the intergroup analysis, regression analysis of pooled data identified PDE as the strongest independent predictor of VO_2max_.

The finding of significantly higher levels of IMCL in the vastus lateralis muscle in the triathlete cohort is in good agreement with the literature. This has previously been described as the athlete's paradox, in which despite a higher amount of IMCL in the skeletal muscles of endurance trained individuals, no impairment in insulin sensitivity was observed (Thamer et al., 2003; Machann et al., 2004). In a sedentary population, increased IMCL has been associated with insulin resistance in human obesity and in T2DM, but endurance-trained athletes have an IMCL content similar to that observed in insulin-resistant obese and T2DM subjects (Dubé et al., 2008). Higher levels of IMCL in the triathlete group are potentially a consequence of the increased need for and efficiency of fatty acid oxidation during long-lasting exercise bouts (Popadic Gacesa et al., 2017).

In the context of higher amounts of IMCL in the skeletal muscle of endurance-trained athletes, together with the indication of enhanced fatty acid oxidation and its importance in maintaining insulin sensitivity, we studied muscle acetylcarnitine levels within our study population. In our study, there was no significant between-group difference. To date, only a few studies have investigated differences in concentrations of acetylcarnitine/carnitine in trained groups of volunteers. But, the link between T2DM and the concentration of acetylcarnitine has been more intensively studied (Mingrone, 2004). Still, slightly higher acetylcarnitine concentrations were observed using biopsy samples from the VL muscle in slow-twitch muscle fibers, probably due to the greater potential for fat oxidation in those types of fibers (Constantin-Teodosiu et al., 1996). This would suggest higher concentrations of acetylcarnitine in the triathlete group. Significantly higher acetylcarnitine concentrations measured by *in vivo* ^1^H-MRS in the vastus lateralis but not in the soleus muscle were recently reported in a group of moderately trained volunteers when compared to normally active population (Klepochová et al., 2017). Lindeboom et al. (2014) did not find a significant difference in acetylcarnitine concentration between endurance-trained and lean sedentary or obese sedentary volunteers and Seiler et al. (2015), in their ^1^H-MRS study with cohort of trained and untrained subjects investigating exercise-induced acetylcarnitine metabolism, did not find differences in acetylcarnitine concentrations in the baseline measurement before exercise, as well. Another important confounder could be the time point of acetylcarnitine measurement in the daily routine and the metabolic conditions of volunteers. A recent study showed significant differences between acetylcarnitine concentrations measured in overnight fasting conditions and metabolite concentrations after lunch (Klepochová et al., 2017). Therefore, it is difficult to compare the results of acetylcarnitine concentration measurements from other studies when the measurements were not recorded at the same time point during the day and/or under the same physical and nutritional conditions. It has already been shown that detection and quantification of acetylcarnitine is challenging (even at 7T) and our data emphasize the need for strict standardization of measurement time, dietary conditions, and also physical activity for the measurement of acetylcarnitine/carnitine. Further studies are necessary to better understand whole-body acetylcarnitine metabolism in humans.

An athletes's involvement in endurance training should stimulate mitochondrial biogenesis (Groennebaek and Vissing, 2017) as a result of training-specific adaptations to a high-volume load compared with sedentary subjects (McCully et al., 1989). This is in agreement with the higher whole body aerobic capacity (VO_2max_), and faster oxidative metabolism demonstrated by ^31^P MRS, in our study.

It is also known that untrained people possess an approximately uniformly distributed muscle fiber spectrum (Staron et al., 2000), while endurance-trained athletes show more slow-twitch fibers in muscles with high oxidative, low glycolytic capacity (Bogdanis, 2012). PCr recovery kinetics can indicate differences in functional mitochondrial performance (Meyer, 1988; Blei et al., 1993; Pesta et al., 2013) and individuals with faster PCr resynthesis typically exhibit greater fatigue-resistance during high-intensity exercise. Our finding of 36% shorter τ_PCr_ is in agreement with Pesta et al. (2013), who reported a 40% faster PCr recovery in endurance-trained athletes than in untrained individuals. Layec et al. (2010) showed faster PCr resynthesis rate and a higher Q_max_ in endurance-trained men, which is also in good agreement with our results, where we found a 77% higher V_PCr_ and 78% higher Q_max_ in triathletes_._ Acidic intracellular pH at the end of exercise could significantly prolong the PCr resynthesis rate (Walter et al., 1997; van den Broek et al., 2007), but we have observed no acidification in either of the groups. The slightly higher pH of triathletes at the end of exercise is in agreement with previous reports on endurance trained subjects (Kent-Braun et al., 1990; Layec et al., 2009) and could potentially suggest faster proton efflux demonstrated earlier in quadriceps of professional road cyclists (Hug et al., 2005).

Carnosine content has been shown to be an indicator of the distribution of muscle fibers since slow-twitch fibers should contain lower concentrations of carnosine than fast-twitch fibers (Baguet et al., 2011). However, we did not find a statistically significant difference between our two groups of volunteers in carnosine content.

Next to the already established and biochemically proven role of specific metabolites (acetylcarnitine, carnosine, PCr, PCr metabolism) in the endurance training-involved adaptation of skeletal muscle metabolism and physiology, our multi-parametric analysis highlighted, again, the link between skeletal muscle PDEs and whole-body metabolism (Szendroedi et al., 2011). Similar to the findings in overweight-to-obese sedentary volunteers (Valkovič et al., 2016), lower concentrations of PDE are linked to higher whole-body and/or skeletal muscle metabolic capacity. Slower PCr recovery rate after exercise alongside increased PDE levels alongside was previously found in the elderly (Waters et al., 2003) patients with lipodystrophy (Sleigh et al., 2012), and hypothyroid patients (Rana et al., 2012). Although the role of PDEs, consisting of GPC and GPE, in the skeletal muscle energy metabolism is not totally clear yet, it is believed to be potentially acting as an indirect measure of oxidative stress on the mitochondrial membrane (Taylor et al., 1997; Schunk et al., 1999). Increased levels of skeletal muscle PDEs have been found also in the patients with Duchenne muscular dystrophy (Kemp et al., 1993a; Hooijmans et al., 2017). Moreover, in a model of membrane defect of Alzheimer's disease Faber et al. reported that an inhibition of oxidative phosphorylation causes accumulation of GPC through accelerated PC turnover (Farber et al., 2000). Rather straightforward and uncomplicated muscle-specific PDE measurement by ^31^P-MRS advocates further detailed and specific studies of this potentially surrogate marker of training and metabolic status. Improved spectral resolution and the observed split into single GPC and GPE signals favors the application of higher field-strength in this regard.

MVC was used in this study as a measure to set the exercise workload, as this takes into account direct power production of the muscle involved in the given exercise. However, one could argue that the endurance component of training status of our triathletes group could, therefore, be less pronounced in our dynamic examinations, as we do not consider the difference in power output connected to the lactate threshold. On the other hand, the measured intracellular pH at the end of exercise did not show substantial acidosis in either of the groups. Using a whole-body metabolism, such as VO_2max_, for exercise load adjustment could pronounce the endurance component in our comparison. However, the dynamic examination would be less specifically calibrated, and thus, would likely become anaerobic, which would contradict our dynamic Pi-to-ATP measurements. It also needs to be mentioned that even under aerobic steady-state exercise conditions, there is still a minor glycolytic component present, which cannot be accounted for under our experimental conditions. However, as the total amount of ATP produced through the glycolysis is negligible in comparison to ATP produced by oxidative phosphorylation, the measured ^31^P parameters can be considered primarily oxidative.

In conclusion, based on this non-invasive ^1^H and ^31^P-MRS study of skeletal muscle metabolic differences between endurance-trained and normally active volunteers, as well as the multivariate analysis of skeletal muscle and whole-body metabolic read-outs, we cannot suggest a single MRS-based parameter as an exclusive biomarker of skeletal muscle fitness and training status that could be, on its own directly usable in sport medicine clinics. It is, rather, a combination of different parameters, such as basal IMCL, acetylcarnitine, and PDE levels as well as measures of PCr metabolism following acute exercise challenge that can be assessed during a single, clinically acceptable multinuclear MRS session. This suggests the need for further broad population cross-sectional and/or focused interventional studies. In these further experiments, the special focus should be placed on the specific role of training regiment, metabolic status, dietary supplementation, and/or therapeutic intervention.

## Ethics statement

This study was carried out in accordance with the recommendations of Ethics Committee with written informed consent from all subjects. All subjects gave written informed consent in accordance with the Declaration of Helsinki. The protocol was approved by The Ethics Committee of the Medical University of Vienna.

## Author contributions

All persons who meet authorship criteria are listed as authors, and all authors certify that they have participated sufficiently in the work to take public responsibility for the content, including participation in the concept, design, analysis, writing, or revision of the manuscript. RK conception and design of study, acquisition of data, analysis and/or interpretation of data, drafting and revising the manuscript, final approval of the version of the manuscript to be published; LV conception and design of study, analysis and/or interpretation of data, drafting the manuscript, final approval of the version of the manuscript to be published; TH conception and design of study, acquisition of data, analysis and/or interpretation of data, final approval of the version of the manuscript to be published; CT: acquisition of data, analysis and/or interpretation of data, final approval of the version of the manuscript to be published; NB, HT, ST, and MiK drafting the manuscript, revising the manuscript critically for important intellectual content, final approval of the version of the manuscript to be published; MaK conception and design of study, analysis and/or interpretation of data, drafting and revising the manuscript critically for important intellectual content, final approval of the version of the manuscript to be published.

### Conflict of interest statement

The authors declare that the research was conducted in the absence of any commercial or financial relationships that could be construed as a potential conflict of interest.
